# Outcomes after minor lower limb amputation for peripheral arterial disease and diabetes: population-based cohort study

**DOI:** 10.1093/bjs/znad134

**Published:** 2023-05-22

**Authors:** Panagiota Birmpili, Qiuju Li, Amundeep S Johal, Eleanor Atkins, Sam Waton, Ian Chetter, Jonathan R Boyle, Arun D Pherwani, David A Cromwell

**Affiliations:** Clinical Effectiveness Unit, Royal College of Surgeons of England, London, UK; Hull York Medical School, Hull, UK; Clinical Effectiveness Unit, Royal College of Surgeons of England, London, UK; Department of Health Services Research and Policy, London School of Hygiene and Tropical Medicine, London, UK; Clinical Effectiveness Unit, Royal College of Surgeons of England, London, UK; Clinical Effectiveness Unit, Royal College of Surgeons of England, London, UK; Hull York Medical School, Hull, UK; Clinical Effectiveness Unit, Royal College of Surgeons of England, London, UK; Hull York Medical School, Hull, UK; Academic Vascular Surgical Unit, Hull University Teaching Hospitals NHS Trust, Hull, UK; Cambridge Vascular Unit, Cambridge University Hospitals NHS Foundation Trust, Cambridge, UK; Staffordshire and South Cheshire Vascular Network, Royal Stoke University Hospital, Stoke-on-Trent, UK; Clinical Effectiveness Unit, Royal College of Surgeons of England, London, UK; Department of Health Services Research and Policy, London School of Hygiene and Tropical Medicine, London, UK

## Abstract

**Background:**

Patients with diabetes and peripheral arterial disease are at increased risk of minor amputation. The aim of study was to assess the rate of re-amputations and death after an initial minor amputation, and to identify associated risk factors.

**Methods:**

Data on all patients aged 40 years and over with diabetes and/or peripheral arterial disease, who underwent minor amputation between January 2014 and December 2018, were extracted from Hospital Episode Statistics. Patients who had bilateral index procedures or an amputation in the 3 years before the study were excluded. Primary outcomes were ipsilateral major amputation and death after the index minor amputation. Secondary outcomes were ipsilateral minor re-amputations, and contralateral minor and major amputations.

**Results:**

In this study of 22 118 patients, 16 808 (76.0 per cent) were men and 18 473 (83.5 per cent) had diabetes. At 1 year after minor amputation, the estimated ipsilateral major amputation rate was 10.7 (95 per cent c.i. 10.3 to 11.1) per cent. Factors associated with a higher risk of ipsilateral major amputation included male sex, severe frailty, diagnosis of gangrene, emergency admission, foot amputation (compared with toe amputation), and previous or concurrent revascularization. The estimated mortality rate was 17.2 (16.7 to 17.7) per cent at 1 year and 49.4 (48.6 to 50.1) per cent at 5 years after minor amputation. Older age, severe frailty, comorbidity, gangrene, and emergency admission were associated with a significantly higher mortality risk.

**Conclusion:**

Minor amputations were associated with a high risk of major amputation and death. One in 10 patients had an ipsilateral major amputation within the first year after minor amputation and half had died by 5 years.

## Teaser

In this population-based cohort study of 22 118 patients undergoing minor amputation for peripheral arterial disease or diabetic complications, the risk of 1-year ipsilateral major amputation was 10.7 per cent; the 1- and 5-year mortality rates were 17.2 and 49.4 per cent respectively. Half of the major amputations occurred in the 3 months after minor amputation. Multiple factors associated with increased risk of amputation and death were identified.

## Introduction

In recent years, there has been an increase in the prevalence of diabetes and peripheral arterial disease (PAD) in the UK and globally, with 1 in 14 people in the UK living with diabetes and 1 in 5 aged over 80 years living with PAD^1–3^. Patients with these conditions are at increased risk of minor amputations, which include removal of toes or parts of the foot up to the ankle. The annual rate of minor amputations has been 22.1 per 10 000 population with diabetes over the past 3 years^4^.

Patients who have a minor amputation are at increased risk of subsequent major amputation (above the ankle), which has a significant impact on mobility and quality of life^5,6^. Therefore, it is important to quantify the risk of limb loss after minor amputation and delineate associated risk factors, in order to tailor surveillance, offer targeted prevention advice to patients, and plan the provision of healthcare resources, with the ultimate aim of minimizing that risk. However, information on long-term outcomes after minor amputation is limited, and the need for a better understanding of how patient conditions can progress has been highlighted as a research priority by patients and clinicians^7,8^. The available information is also difficult to interpret. Studies have reported diverse estimated rates of repeat amputations (at the same or higher level after an initial minor amputation), ranging from 17 to 35 per cent for the ipsilateral and from 8 to 15 per cent for the contralateral limb, and the time frame of reported outcomes was not consistently specified^5–12^.

The aim of this study was to estimate the rate of subsequent ipsilateral and contralateral major and minor amputation and death after an initial minor lower limb amputation, and to identify factors that increase this risk, to inform patient counselling, future research, disease prevention strategies, and healthcare planning.

## Methods

This observational cohort study used a linked dataset that comprised the pseudoanonymized patient records from Hospital Episode Statistics (HES) Admitted Patient Care (APC), the Office for National Statistics (ONS) Death Registry, and the National Vascular Registry (NVR). The HES APC is an administrative database that contains information about all day-case and inpatient admissions to National Health Service (NHS) hospitals in England^13^, with diagnoses documented using ICD-10^14^ and procedures described using OPCS-4^15^. The ONS Death Registry holds the date and cause of death for English residents^16^. The NVR is a national clinical audit that collects information on five major vascular procedures performed in UK NHS vascular units, including revascularizations and amputations. The data linkage was performed by NHS Digital, and patients were given a unique identifier that allowed their hospital care to be followed over time. The study involved secondary analysis of existing pseudoanonymized data and was exempt from UK national ethics committee approval.

### Study population

The study cohort included all patients aged 40 years and over with PAD or diabetes, who underwent a minor lower limb amputation between 1 January 2014 and 31 December 2018 in England, with data extracted from the HES database. HES was used to identify the population of interest, as minor amputations are not captured well in the NVR and the case ascertainment is very poor. The first minor amputation each patient underwent in the study interval was considered as the index procedure, and the admission during which it was performed the index admission. Minor amputation was defined as any amputation through or below the ankle joint (OPCS codes X10, X11), and major amputation as any amputation above the ankle joint (X09). Patients who had a record of any amputation in the 3 years before the index procedure were excluded. The 3-year cut-off was based on data availability. Also excluded were non-English residents, patients with incomplete records (missing data on age, sex, area of residence, admission mode, amputation date or side), and patients whose primary indication for minor amputation was trauma, cancer, or musculoskeletal or connective tissue disorders, when the latter were treated by trauma and orthopaedic surgeons (*Table S1*). Finally, patients with bilateral minor amputations as the index event were excluded, as it was not possible to categorize previous revascularization as performed or not when only one of the limbs was operated on.

### Patient characteristics

Patient characteristics related to the index admission and used in the analysis were: age, sex, presence of diabetes, PAD, selected comorbidities, frailty score, and socioeconomic status. Age was categorized into four intervals (40–59, 60–69, 70–79, 80 or more years), each containing a similar proportion of patients. The presence of diabetes and PAD was determined from the relevant ICD-10 codes occurring in any diagnosis field in the index hospital admission and admissions in the 3 years before that^17^ (*Table S1*).

Frailty was assessed using the Secondary Care Administrative Records Frailty (SCARF) index, which is based on the cumulative deficit model of frailty, and comprises 32 deficits that cover medical comorbidities and functional impairment^18^. The Royal College of Surgeons Charlson Comorbidity Index score was used to measure comorbidity burden (excluding PAD and diabetes), and was calculated using ICD-10 diagnostic codes over 2 years^19^. Socioeconomic status was measured using the Index of Multiple Deprivation 2019, based on the patient’s area of residence, with the areas divided into quintiles, from least to most deprived^20^.

Open and endovascular revascularization procedures were identified in the 3 years before the minor amputation using OPCS codes (*Table S1*). Ipsilateral revascularization was considered as any attempt at same-side revascularization before or within 20 days after the index minor amputation, to identify patients who required an intervention to improve blood flow before or at the time of the minor amputation. The 20-day cut-off time was chosen as the daily revascularization rate was high and likely related to the indication for minor amputation. When missing, the side of revascularization was considered to be the same as the amputation side. Information on type of admission (emergency *versus* elective), level of amputation (foot *versus* toe), and presence of gangrene or osteomyelitis was also collected from the index admission record. The type of amputations, coded as foot and toe, are provided in *Table S1*. Date, side, and level of subsequent amputations were also recorded. The linked NVR dataset was used to ascertain the side of major amputation and revascularization procedures if these data were missing from the HES dataset, as these procedures are captured well in the NVR with high case ascertainment.

### Outcomes

The primary outcomes were ipsilateral major amputation and death after the index minor amputation. Secondary outcomes included ipsilateral minor re-amputations, and contralateral minor and major amputations. If patients had more than one re-amputation, the first amputation of each type (ipsilateral, contralateral, minor, major) was considered for each outcome. Survival times were estimated from the time of the procedure and used a patient’s date of death from the ONS Death Registry. The end of follow-up was defined as 31 December 2020, giving a minimum follow-up of 2 years.

### Statistical analysis

A complete-case analysis was undertaken. Categorical variables are expressed as numbers with percentages. Time-to-event data (time to amputation, time to death) are summarized as median (i.q.r.) values. The significance of differences in the time-to-event curves between patient groups was evaluated using the log rank test.

Flexible parametric competing-risks survival analysis was carried out to identify factors associated with the time to ipsilateral major amputation and survival^21^. The flexible parametric model uses restricted cubic splines to model the underlying shape of the hazard function and time-dependent effects on the log-cumulative hazard scale^22,23^. The competing-risks framework was chosen because patients were simultaneously at risk of major amputation and death, and death precluded the occurrence of major amputation. Cause-specific cumulative incidence functions were estimated for each outcome^24^, which give the probability of experiencing an event after a specific time.

Explanatory variables included in the model were age, sex, presence of PAD and diabetes, SCARF index score, Charlson Comorbidity Index score, deprivation, type of admission, type of index minor amputation, previous/concurrent revascularization procedure, and presence of gangrene or osteomyelitis. The Bayesian information criterion was used to determine the best fitting model, and select the appropriate cubic splines for the baseline hazard function and the time-dependent effects. In the model for the risk of major amputation, time-dependent effects were included for age, type of admission, and type of index minor amputation because the proportional hazards assumption was not met; in the model for death, mode of admission was included as a time-dependent effect.

All statistical tests were two-sided and *P* < 0.050 was considered statistically significant. All analyses were performed in Stata^®^ version 17 (StataCorp, College Station, TX, USA)^25^ with the command stpm2cr used for the competing-risk analysis. Results are presented in accordance with the RECORD extension of the STROBE statement for observational studies^26^.

## Results

Data on some 35 707 patients who underwent minor amputations between 2014 and 2018 were extracted from HES. Of these, 9175 were excluded because they did not have a diagnosis of PAD or diabetes. Also excluded were 1962 patients who had previous amputations within 3 years of the index procedure, 1057 who were aged less than 40 years, 758 who had missing information on covariates, 183 who were non-English residents and for whom information on social deprivation was therefore missing, and 454 who had bilateral index minor amputations.

The study cohort for analysis comprised 22 118 patients. The majority were men (16 808, 76.0 per cent) and had diabetes (18 473, 83.5 per cent). The index minor amputation was more often performed after an emergency admission (14 335, 64.8 per cent), and 4002 (18.1 per cent) were foot amputations (*Table 1*).

**Table 1:** Baseline characteristics of patients who underwent minor amputation for peripheral arterial disease or diabetes mellitus in English NHS hospitals between 2014 and 2018

	Total (*n* = 22 118)	PAD and diabetes (*n* = 10 823)	PAD only (*n* = 3645)	Diabetes only (*n* = 7650)
**Age (years)**				
40–59	5987 (27.1)	2350 (21.7)	462 (12.7)	3175 (41.5)
60–69	5759 (26.0)	3017 (27.9)	734 (20.1)	2008 (26.3)
70–79	5829 (26.4)	3206 (29.6)	1097 (30.1)	1526 (20.0)
≥ 80	4543 (20.5)	2250 (20.8)	1352 (37.1)	941 (12.3)
Sex ratio (M : F)	16 808 : 5310	8464 : 2359	2531 : 1114	5813 : 1837
Previous revascularization	9282 (42.0)	6542 (60.5)	2535 (69.6)	205 (2.7)
**SCARF index score**				
Fit	106 (0.5)	< 5 (0)	38 (1.0)	66 (0.9)
Mild frailty	1494 (6.8)	219 (2.0)	485 (13.3)	790 (10.3)
Moderate frailty	7127 (32.2)	2650 (24.5)	1234 (33.9)	3243 (42.4)
Severe frailty	13 391 (60.5)	7952 (73.5)	1888 (51.8)	3551 (46.4)
**RCS Charlson Comorbidity Index score**				
0	8287 (37.5)	3283 (30.3)	1078 (29.6)	3926 (51.3)
1	6197 (28.0)	3041 (28.1)	1102 (30.2)	2054 (26.9)
2	3887 (17.6)	2149 (19.9)	765 (21.0)	973 (12.7)
3	3747 (16.9)	2350 (21.7)	700 (19.2)	697 (9.1)
**Deprivation**				
1 (least deprived)	2893 (13.1)	1409 (13.0)	506 (13.9)	978 (12.8)
2	3724 (16.8)	1788 (16.5)	695 (19.1)	1241 (16.2)
3	4546 (20.6)	2208 (20.4)	737 (20.2)	1601 (20.9)
4	4884 (22.1)	2407 (22.2)	759 (20.8)	1718 (22.5)
5 (most deprived)	6071 (27.4)	3011 (27.8)	948 (26.0)	2112 (27.6)
Emergency admission	14 335 (64.8)	7270 (67.2)	2050 (56.2)	5015 (65.6)
Index foot amputation	4002 (18.1)	2123 (19.6)	688 (18.9)	1191 (15.6)
Osteomyelitis	8086 (36.6)	3515 (32.5)	679 (18.6)	3892 (50.9)
Ulcer	12 807 (57.9)	6323 (58.4)	1498 (41.1)	4986 (65.2)
Gangrene	9038 (40.9)	4558 (42.1)	1451 (39.8)	3029 (39.6)

Values are *n* (%). SCARF, Secondary Care Administrative Records Frailty; RCS, Royal Collage of Surgeons.

### Ipsilateral major amputations

During the study interval, 3118 patients (14.1 per cent) underwent ipsilateral major amputation after the index minor amputation; there were 2332 (74.8 per cent) below-knee, 695 (22.3 per cent) above-knee, and 91 (2.9 per cent) through-knee procedures. The median time from minor to major amputation was 76 (i.q.r. 20–344) days; it was significantly shorter for below-knee than above-knee amputations (median 65 (19–300) *versus* 113 (27–469) days; *P* < 0.001). Patients with diabetes without PAD had a significantly longer time to major amputation (median 164 (26–669) days) than those with diabetes and PAD (60 (19–268) days), or no diabetes (91 (21–309) days) (*P* < 0.001).

**Fig. 1:**
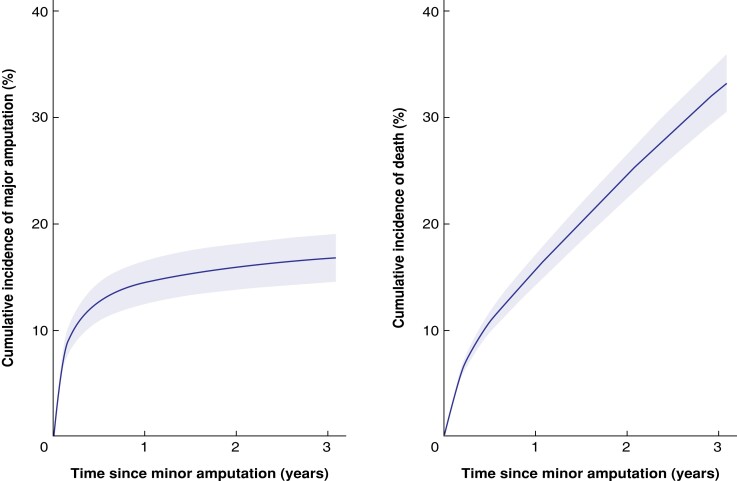
Risk of ipsilateral major amputation and death after minor amputation for a typical person in the study cohort **a** Major amputation and **b** death. Charateristics of typical study participant: 70–79-year-old man, with diabetes and peripheral arterial disease, no other comorbidities, severe frailty, highest deprivation quintile, admitted as emergency with gangrene, with no previous revascularization, having toe amputation.

**Fig. 2:**
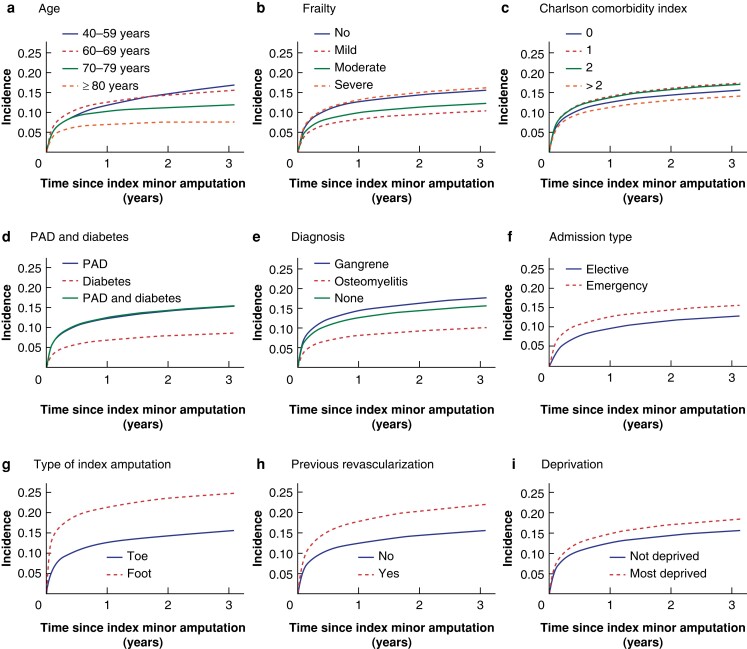
Cumulative incidence of ipsilateral major amputation over time after index minor amputation in relation to demographic and clinical factors **a** Age, **b** frailty, **c** Charlson Comorbidity Index score, **d** peripheral arterial disease (PAD) and diabetes, **e** diagnosis, **f** admission type, **g** type of index amputation, **h** previous revascularization, and **i** deprivation. The 60–69-years age group, presence of PAD and diabetes, and emergency admission were used as baseline for all graphs apart from those depicting these variables.

The estimated rate of ipsilateral major amputations was 10.7 (95 per cent c.i. 10.3 to 11.1) per cent at 1 year and 13.3 (12.8 to 13.7) per cent at 3 years (*Table 2*). *Figure 1* shows the cumulative incidence of major amputation over time for a typical person in the study cohort. After adjustment, the rate of subsequent ipsilateral major amputation was higher for emergency admissions, patients with index foot amputations, those who had previous or concurrent revascularization, and patients in the highest deprivation quintile (*Fig. 2*). It was also higher for men, patients with severe frailty, and those with a diagnosis of gangrene. Additionally, patients with PAD with or without diabetes had a higher risk of undergoing an ipsilateral major amputation than those with diabetes alone (respectively HR 1.89, 95 per cent c.i. 1.69 to 2.12; and HR 1.84, 1.61 to 2.12) (*Table S2*).

**Table 2:** Estimated rate of major amputation, further minor amputation, and death at 1-, 3-, and 5-year follow-up, by type of amputation

	Follow-up		
	1 year	3 years	5 years
Ipsilateral minor amputation (%)	24.85 (24.18, 25.52)	32.36 (31.63, 33.10)	36.06 (35.24, 36.87)
Ipsilateral major amputation (%)	10.69 (10.29, 11.09)	13.26 (12.82, 13.70)	14.57 (14.09, 15.05)
Contralateral minor amputation (%)	6.86 (6.45, 7.27)	16.86 (16.22, 17.50)	23.19 (22.34, 24.03)
Contralateral major amputation (%)	1.89 (1.72, 2.06)	4.76 (4.48, 5.04)	6.71 (6.34, 7.07)
Death (%)	17.22 (16.74, 17.70)	35.23 (34.61, 35.84)	49.36 (48.60, 50.12)

Values in parentheses are 95% confidence intervals.

### Mortality

**Fig. 3:**
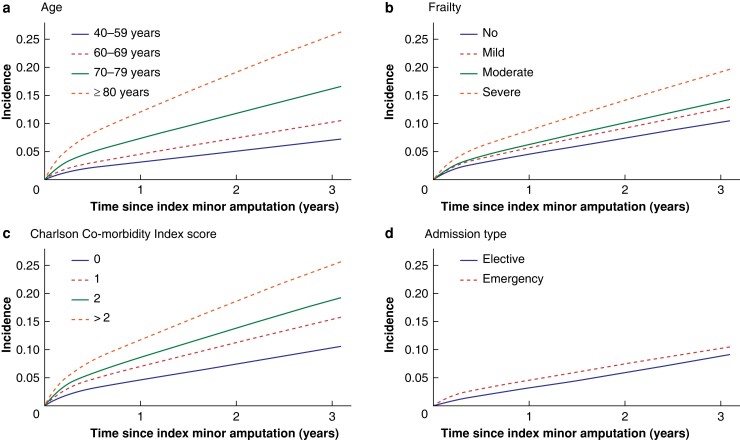
Cumulative incidence of death over time after index minor amputation in relation to demographic and clinical factors **a** Age, **b** frailty, **c** Charlson Comorbidity Index score, and **d** admission type, The 60–69-years age group, presence of peripheral arterial disease and diabetes, and emergency admission were used as baseline for all graphs apart from those depicting these variables.

By the end of the study interval, 8923 patients (40.3 per cent) had died without limb loss, and 1623 (7.3 per cent) had died after undergoing an ipsilateral major amputation. The main causes of death are summarized in *Table S3*. The estimated mortality rate after minor amputation was 17.2 (95 per cent c.i. 16.7 to 17.7) per cent at 1 year and 35.2 (34.6 to 35.8) per cent at 3 years; the mortality rate at 5 years was almost 50 per cent (49.4 (48.6 to 50.1) per cent). The median time to death was 19 months (584 (i.q.r. 191–1083) days). *Figure 1* shows the cumulative mortality rate over time for a typical person in the study cohort. Older age, severe frailty, more comorbidities, admission as an emergency (*versus* elective), presentation with gangrene, absence of previous revascularization, and presence of PAD with or without diabetes (*versus* diabetes alone) were associated with a significantly higher risk of death (*P* < 0.001 for all factors) (*Fig. 3*). With increasing age and frailty, patients were less likely to undergo major amputation and had a higher mortality risk (*Fig. S1*).

### Other subsequent minor and contralateral major amputations

Patients had a 24.9 (95 per cent c.i. 24.2 to 25.5) per cent chance of having another ipsilateral minor amputation and a 6.9 (6.5 to 7.3) per cent chance of having a contralateral minor amputation if they were alive and without major amputation at 1 year after the initial minor amputation. The estimated rates of ipsilateral minor re-amputation and contralateral amputations at 1-, 3-, and 5-years of follow-up are summarized in *Table 2*. Median time to subsequent ipsilateral minor amputation was 114 (i.q.r. 30–410) days and time to contralateral minor amputation was 556 (263–956) days.

## Discussion

In this population-based cohort study, the estimated ipsilateral major amputation rate was 10.7 per cent in the first year after minor amputation for PAD or complications of diabetes, and factors associated with increased risk were identified. The estimated mortality rate was 17.2 (95 per cent c.i. 16.7 to 17.7) per cent at 1 year and 49.4 (48.6 to 50.1) per cent at 5 years after minor amputation, similar to rates reported in other large studies^27^. Older age, severe frailty, more comorbidities, presentation with gangrene, and emergency admission were associated with a significantly higher risk of death.

These findings add to previous results produced by other studies. *Littman et al.*^9^ reported a similar ipsilateral major amputation rate of 10.2 per cent and ipsilateral minor amputation rate of 23.9 per cent at 1 year after initial toe amputation in a study using the US Veteran Health Administration database. However, that study included only toe amputations on patients with diabetes and had a younger population overall, which may explain the lower 1-year mortality rate than in the present analysis (12.2 *versus* 17.2 per cent).

These results are also comparable to those in studies of patients undergoing minor amputation, irrespective of diabetes status. A study^10^ using a cohort from a single US centre (1998–2010) reported similar rates of ipsilateral major amputation of 10.5 per cent, and contralateral minor and major amputation rates of 7.0 and 3.2 per cent respectively, at 1 year. However, a much higher rate of ipsilateral repeat minor amputations of 24.9 per cent was identified in the present study, compared with 14.2 per cent^10^, which may be attributed to the fact that the competing risks of death and major amputation were taken into account when estimating the risk of minor amputation.

A further US study^5^ using data between 2005 and 2013 also found that the rate of subsequent major and minor amputations was highest in patients with PAD and diabetes (6.3 and 16 per cent respectively), compared with rate among those with only one of the conditions, with an overall major amputation rate of 5.1 per cent and repeat minor amputation rate of 14.5 per cent. However, these rates were not provided for specific follow-up times, and so comparisons are difficult. The laterality of the procedures was also not reported, so it is unclear whether the study considered all re-amputations or only ipsilateral ones.

Previous revascularization attempts have not been frequently examined as a variable in studies of minor amputation. Previous or concurrent revascularization was independently associated with an increased risk of major amputation (HR (*versus* no revascularization) 1.46, 95 per cent c.i. 1.34 to 1.59), which may indicate more complex or advanced atherosclerotic disease that required an intervention to improve blood flow to the foot. However, the risk of death was slightly lower for these patients, after controlling for other factors, which was not anticipated (HR 0.91, 0.86 to 0.96).

More than one-third of patients who underwent minor amputation in this study (34.6 per cent) had diabetes without a code for PAD. Apart from the possibility of coding errors, this information may reflect the presence of distal PAD in the foot that was either not identified on standard diagnostic testing not amenable to revascularization, rather than the absence of vascular disease overall^28^. Additionally, 50.9 per cent of patients with diabetes but no PAD had osteomyelitis, which highlights the role of infection as a possible cause of minor amputation in this group. Conversely, having PAD codes in the patient record most likely indicates the presence of macrovascular disease^29,30^. Patients with PAD without diabetes had a higher risk of undergoing an ipsilateral major amputation after an index minor amputation than patients with diabetes alone. Lower amputation rates have previously been associated with higher intensity of vascular care^31^ and foot care provision^32^ for patients with diabetes. However, patients with PAD without diabetes do not have routine access to foot care services, and these results suggest there may be benefit in expanding access to specialist foot care services to all patients with foot wounds due to PAD or diabetes. This recommendation has also been made by the National Wound Care Strategy Programme^33^, an initiative commissioned by NHS England and NHS Improvement, aiming to improve care for people with lower limb wounds. Despite these indications, there is a lack of high-quality evidence on whether having intensive foot care services for patients with PAD (without diabetes) might lower subsequent amputation rates or reduce the risk of adverse outcomes; research is required in this area. A preventive strategy that has been found to decelerate the progression of disease is risk factor modification, in the form of smoking cessation, aggressive glycaemic control, lipid control, BP control, and antiplatelet therapy^34^.

This study showed that patients with PAD and diabetes had shorter times to major amputation than those with PAD alone or diabetes alone. This information can be used to tailor the follow-up intervals of patients after minor amputation, with more intensive observation for the first 3 months, as 1647 patients (53 per cent) had a subsequent major amputation during that time.

The study has several strengths, such as the large size of the cohort and the inclusion of patients with and without diabetes. Additionally, the data described recent clinical practice and explored a relatively long follow-up. Moreover, the competing-risks framework permitted separate estimation of the risk of major amputation, death, and of subsequent minor amputation. However, the study has several limitations. First, the data source was primarily an administrative database and so there is risk of error owing to inaccurate coding or the omission of clinical information by hospital coders^35,36^. Second, it was not possible to account for the severity of PAD and other potentially relevant variables, such as smoking and ethnicity, as these are not well documented in the ICD-10 diagnosis codes used in HES. However, the selection of ICD-10 codes used to define the cohort and the side of the procedure were informed by a previous study^37^ comparing coding between HES and NVR databases.

## Supplementary Material

znad134_Supplementary_Data

## Data Availability

The data governance arrangements for the study do not allow the authors to redistribute NVR or HES data to other parties. Researchers interested in using NVR data can apply for access through Health Quality Improvement Partnership’s Data Access Request facility (https://www.hqip.org.uk). Researchers can apply for access to HES data through NHS Digital processes.
