# Supplementary material for: Outcomes after minor lower limb amputation for peripheral arterial disease and diabetes: population-based cohort study
Source: Br J Surg. 2023 May 22;110(8):958–65. doi: 10.1093/bjs/znad134 (PMC10361679; doi:10.1093/bjs/znad134)
Supplement: znad134_Supplementary_Data [file znad134_supplementary_data.docx]

**Outcomes after minor lower limb amputation for peripheral arterial disease and diabetes: population-based cohort study**

Panagiota Birmpili^1,2^, Qiuju Li^1,3^, Amundeep S Johal^1^, Eleanor Atkins^1,2^, Sam Waton^1^, Ian Chetter^2,4^, Jonathan R Boyle^5^, Arun D Pherwani^6^, David A Cromwell^1,3^

^1^Clinical Effectiveness Unit, Royal College of Surgeons of England, London, UK

^2^Hull York Medical School, Hull, UK

^3^Department of Health Services Research and Policy, London School of Hygiene and Tropical Medicine, London, UK

^4^Academic Vascular Surgical Unit, Hull University Teaching Hospitals NHS Trust, Hull, UK

^5^Cambridge Vascular Unit, Cambridge University Hospitals NHS Foundation Trust, Cambridge, UK

^6^Staffordshire & South Cheshire Vascular Network, Royal Stoke University Hospital, Stoke-on-Trent, UK

Corresponding author:

Panagiota Birmpili, Clinical Effectiveness Unit, The Royal College of Surgeons of England, 38-43 Lincoln's Inn Fields, Holborn, London, WC2A 3PE, UK.

Email: [Panagiota.Birmpili@nhs.net](mailto:Panagiota.Birmpili@nhs.net); ORCID ID: [0000-0002-0108-1733](https://orcid.org/0000-0002-0108-1733); Twitter: @pennybir

**Supplementary Materials - Index**

| **Supplementary Figures and Tables** |  |
| --- | --- |
| Table S1. OPCS and ICD-10 codes used in the study | *pag. 2* |
| Table S2. Coefficients table for the competing risks of ipsilateral major amputation and death. The coefficients for the splines and the time effects have been omitted. | *pag. 3* |
| Table S3. Causes of death of patients that underwent minor amputations | *pag. 4* |
| Figure S1. Stacked cumulative incidence of ipsilateral major amputation and death following minor amputation by age group and frailty. Presence of PAD and diabetes and emergency admission were used as baseline. | *pag. 5* |
| **References** | *pag. 6* |

**Supplementary Figures and Tables**

Table S1. OPCS and ICD-10 codes used in the study

| **Condition** | **ICD-10 Codes^1^** |
| --- | --- |
| Diabetes | E10, E11, E12, E13, E14 |
| Diabetes with peripheral circulatory complications | E105, E115, E135, E145 |
| Peripheral arterial disease | I70x, I73x, I74x, I77x, I78x, I79x |
| Cellulitis | L03x, L08x |
| Osteomyelitis | M86x |
| Ulcer | L89x, L984, L97x |
| Gangrene | R02x |
|  |  |
| Excluded diagnoses: |  |
| Cancer | C00x-C99x |
| Trauma | S7-9 (apart from S90-91), T0-7, T9, V, W, X |
| Musculoskeletal if treated by T&O | M (apart from M86), Q6, Q7 |
|  |  |
| **Procedure** | **OPCS Codes^2^** |
| Revascularisation | L161, L162, L163, L206, L216, L501, L502, L503, L504, L505, L506, L511, L512, L513, L514, L515, L516, L518, L519, L521, L522, L528, L529**,** L531, L538, L539, L581, L582, L583, L584, L585, L586, L587, L588, L589, L591, L592, L593, L594, L595, L596, L597, L598, L599, L601, L602, L603, L604, L608, L609, L621, L652, L653, L681, L682, L541, L544, L631, L635, L662, L665, L667, L711 |
| Major lower limb amputation | X09x |
| Minor foot amputation | X101 Amputation of foot through ankle X102 Disarticulation of tarsal bones X103 Disarticulation of metatarsal bones X104 Amputation through metatarsal bones X108 Other specified X109 Unspecified |
| Minor toe amputation | X111 Amputation of great toe X112 Amputation of phalanx of toe X118 Other specified X119 Unspecified |

Table S2. Coefficients table for the competing risks of ipsilateral major amputation and death. The coefficients for the splines and the time effects have been omitted.

|  | **Ipsilateral major amputation** | | **Mortality** | |
| --- | --- | --- | --- | --- |
|  | HR (95% CI) | p-value | HR (95% CI) | p-value |
| **Age** |  | <0.001 |  | <0.001 |
| 40-59 | 1.01 (0.91 - 1.11) |  | 0.68 (0.62 - 0.74) |  |
| 60-69 | 1 |  | 1 |  |
| 70-79 | 0.77 (0.70 - 0.85) |  | 1.63 (1.52 - 1.75) |  |
| >=80 | 0.51 (0.45 - 0.57) |  | 2.75 (2.57 - 2.94) |  |
| **Female gender** | 0.90 (0.83 - 0.99) | 0.022 | 1.03 (0.98 - 1.09) | 0.239 |
| **PAD - Diabetes** |  | <0.001 |  | <0.001 |
| Diabetes only | 1 |  | 1 |  |
| PAD only | 1.84 (1.61 - 2.12) |  | 1.16 (1.07 - 1.25) |  |
| PAD and Diabetes | 1.89 (1.69 - 2.12) |  | 1.19 (1.11 - 1.27) |  |
| **Emergency admission** | 1.37 (1.26 - 1.48) | <0.001 | 1.39 (1.31 - 1.47) | <0.001 |
| **Index foot amputation** | 1.84 (1.70 - 1.99) | <0.001 | 0.97 (0.92 - 1.03) | 0.362 |
| **Previous revascularisation** | 1.46 (1.34 - 1.59) | <0.001 | 0.91 (0.86 - 0.96) | <0.001 |
| **Most deprived** | 1.20 (1.11 - 1.29) | <0.001 | 1.02 (0.97 - 1.08) | 0.403 |
| **Gangrene** | 1.15 (1.07 - 1.23) | <0.001 | 1.11 (1.06 - 1.16) | <0.001 |
| **Osteomyelitis** | 0.63 (0.58 - 0.69) | <0.001 | 1.03 (0.98 - 1.08) | 0.241 |
| **Scarf Frailty Index** |  | <0.001 |  | <0.001 |
| Fit | 1 |  | 1 |  |
| Mild frailty | 0.65 (0.34 - 1.23) |  | 1.25 (0.68 - 2.29) |  |
| Moderate frailty | 0.78 (0.42 - 1.45) |  | 1.39 (0.77 - 2.52) |  |
| Severe frailty | 1.05 (0.56 - 1.96) |  | 1.98 (1.09 - 3.58) |  |
| **RCS Charlson score** |  | <0.001 |  | <0.001 |
| 0 | 1 |  | 1 |  |
| 1 | 1.13 (1.02 - 1.25) |  | 1.55 (1.44 - 1.67) |  |
| 2 | 1.10 (0.98 - 1.24) |  | 1.93 (1.78 - 2.10) |  |
| 3 | 0.90 (0.79 - 1.02) |  | 2.67 (2.46 - 2.89) |  |

Table S3. Causes of death of patients that underwent minor amputations

| **Cause of death** | **ICD-10 code^1^** | **n** | **%** |
| --- | --- | --- | --- |
| Sepsis | A40, A41 | 831 | 7.9% |
| Infectious conditions | A04, A09, A17, A31, A49, B02, B49, B99, L03, L08, J10, J11 | 53 | 0.5% |
| Lung cancer | C34 | 191 | 1.8% |
| Non-lung neoplasms | C00-D48 (not C34) | 651 | 6.2% |
| Diabetes | E10, E11, E14, E16, R73 | 79 | 0.7% |
| Metabolic and electrolyte disorders | E66, E83-E88 | 31 | 0.3% |
| Nervous system conditions/ dementia | F01 ,F03, G00, G04, G12, G20, G23,  G30-G41, G61, G70, G93, G96 | 287 | 2.7% |
| Acute myocardial infarction | I21, I23, I24 | 749 | 7.1% |
| Chronic ischaemic heart disease | I25 | 675 | 6.4% |
| Pulmonary embolism | I26 | 100 | 0.9% |
| Cardiac arrest | I46 | 128 | 1.2% |
| Heart failure | I50 | 948 | 9.0% |
| Other heart conditions | I05, I11-I13, I27-I45, 147-I49, I51,I95-I99 | 258 | 2.4% |
| Intracranial injury/haemorrhage | I60, I61, I62, S06 | 126 | 1.2% |
| Stroke | I63, I64 | 228 | 2.2% |
| Other cerebrovascular conditions | I67 | 24 | 0.2% |
| Vascular conditions | I70-I74, I77, R02, K55 | 431 | 4.1% |
| Pneumonia | J12, J13, J15, J18, J20, J22 | 1659 | 15.7% |
| COPD | J43, J44, J47 | 90 | 0.9% |
| Aspiration pneumonia | J69 | 267 | 2.5% |
| Respiratory failure, ARDS | J80, J81, J96 | 201 | 1.9% |
| Other respiratory conditions | J84, J86, J90-J95, J98, R06, R09, T17, T71 | 140 | 1.3% |
| Gastrointestinal conditions | K22-K66, R19 | 88 | 0.8% |
| Liver conditions | K70-K76, K81-K83 | 113 | 1.1% |
| Ulcer of lower limb | L97 | 23 | 0.2% |
| Osteomyelitis | M86 | 42 | 0.4% |
| Renal conditions | N00, N04, N17-N19, N28, N30, N39 | 587 | 5.6% |
| Haemorrhage (GI/Other) | R04, R58, K92, I85 | 90 | 0.9% |
| Old age | R54 | 227 | 2.2% |
| Shock | R57 | 53 | 0.5% |
| Other symptoms | R68, R99 | 697 | 6.6% |
| Complications of procedure | T80-T85, T87 | 126 | 1.2% |
| COVID | U07 | 229 | 2.2% |
| Miscellaneous |  | 98 | 0.9% |
| Missing |  | 26 | 0.2% |

Figure S1. Stacked cumulative incidence of ipsilateral major amputation and death following minor amputation by age group and frailty. Presence of PAD and diabetes and emergency admission were used as baseline.


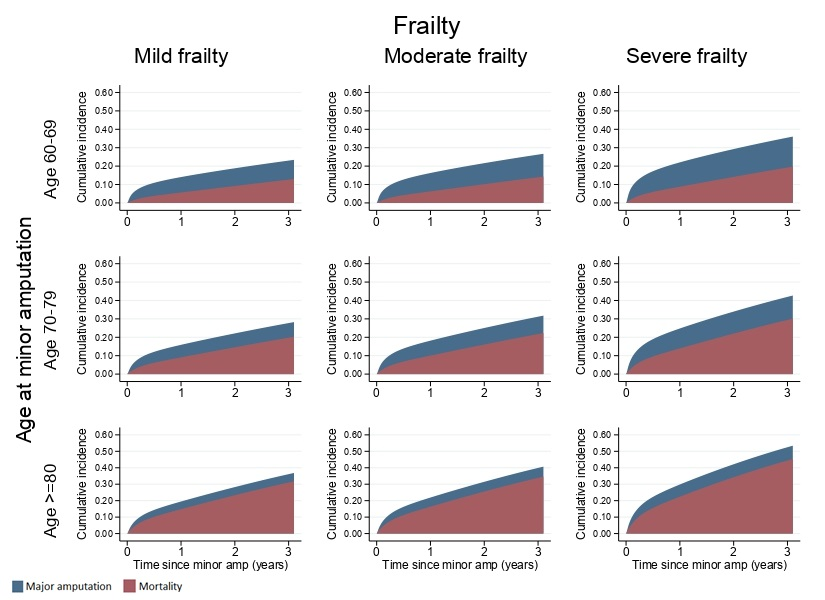


**References**

1 NHS Digital. National Clinical Coding Standards ICD-10 5th Edition. Leeds: NHS Digital; 2021.

2 NHS Digital. National Clinical Coding Standards OPCS-4.8 Reference Book. Leeds: NHS Digital; 2017.
